# Design of a multifiber light delivery system for photoacoustic-guided surgery

**DOI:** 10.1117/1.JBO.22.4.041011

**Published:** 2017-01-13

**Authors:** Blackberrie Eddins, Muyinatu A. Lediju Bell

**Affiliations:** a Johns Hopkins University, Department of Biomedical Engineering, Baltimore, Maryland 21218, United States; b Johns Hopkins University, Department of Electrical and Computer Engineering, Baltimore, Maryland 21218, United States

**Keywords:** interventional photoacoustic imaging, image-guided surgery, specialized light delivery, transcranial imaging

## Abstract

This work explores light delivery optimization for photoacoustic-guided minimally invasive surgeries, such as the endonasal transsphenoidal approach. Monte Carlo simulations were employed to study three-dimensional light propagation in tissue, comprising one or two 4-mm diameter arteries located 3 mm below bone, an absorbing metallic drill contacting the bone surface, and a single light source placed next to the 2.4-mm diameter drill shaft with a 2.9-mm diameter spherical drill tip. The optimal fiber distance from the drill shaft was determined from the maximum normalized fluence to the underlying artery. Using this optimal fiber-to-drill shaft distance, Zemax simulations were employed to propagate Gaussian beams through one or more 600 micron-core diameter optical fibers for detection on the bone surface. When the number of equally spaced fibers surrounding the drill increased, a single merged optical profile formed with seven or more fibers, determined by thresholding the resulting light profile images at 1/e times the maximum intensity. We used these simulations to inform design requirements, build a one to seven multifiber light delivery prototype to surround a surgical drill, and demonstrate its ability to simultaneously visualize the tool tip and blood vessel targets in the absence and presence of bone. The results and methodology are generalizable to multiple interventional photoacoustic applications.

## 1 Introduction

Photoacoustic imaging has the potential to enable real-time visualization of regions of interest during surgery. This is significant because it is more difficult to perform a surgery with static reference images (e.g., computed tomography scans and magnetic resonance images) of internal structures, though surgeons typically use these kinds of images to visualize targets hidden by bone and other tissues. Although ultrasound imaging provides real-time images of internal structures, it is often difficult to deliver miniature probes to the surgical site without sacrificing image quality (e.g., resolution). For these and other reasons, several researchers are investigating interventional photoacoustic systems.

Most applications of interventional photoacoustics require utilization of an optical fiber. The most straightforward method to integrate an optical fiber is to couple a bare fiber to a pulsed laser and detect signals with an external ultrasound probe, which was the method used in *ex vivo* pilot studies to discriminate nerves from tendons^1^ and localize blood vessels hidden by bone.^2^ Another approach is to nest the fiber inside a hollow needle, as implemented to explore photoacoustic-guided biopsy techniques^3^ and to visualize brachytherapy seeds inside *in vivo* prostates.^4^ Microscopic applications utilize a more conventional approach by integrating the light delivery system with a single ultrasound transducer element to receive acoustic signals, enabling tasks such as the evaluation of both intraoperative breast tumor margins^5^ and intravascular positions of stents and plaque.^6^ One common feature of these and other potential interventional applications is that they utilize a single optical fiber. Although multifiber light delivery systems have previously been designed to surround ultrasound probes^7^^,^^8^ and to illuminate tissue for direct registration of photoacoustic images to stereo camera images,^9^ to the authors’ knowledge, no light delivery systems exist to surround surgical tools.

Our group is exploring multifiber light delivery systems to surround surgical tools, with applications to minimally invasive surgery, such as neurosurgeries to remove pituitary tumors using the endonasal transsphenoidal approach. In this approach, the light delivery system would be attached to the surgical tool, which is inserted in the nose and would transmit light across the sphenoid bone. The internal carotid arteries hidden behind the bone would absorb the light, undergo thermal expansion, and generate an acoustic response to be detected by an external transcranial ultrasound probe placed on the patient’s temple.^2^ The minimum energy required to visualize real blood ranged from 1.2 to 6 mJ when the thickness of the cranial bone ranged from 0 to 2 mm, which corresponds to a fluence range of 4 to $21\;\mathrm{mJ}/{\mathrm{cm}}^{2}$ for the 6-mm diameter fused fiber bundle used to deliver the light.^10^ These results demonstrated the feasibility of visualizing real blood in the presence of bone within the $26.4\;\mathrm{mJ}/{\mathrm{cm}}^{2}$ safety limit for 760-nm wavelength light.^11^ In addition, placement of a mock tool tip (consisting of a metal ball glued to a paper clip) provided satisfactory preliminary evidence that surgical tool tips can be visualized simultaneously with blood vessels using a single 6-mm diameter fused fiber bundle.^10^

Although previous results are encouraging, the light delivery design has limited practicality for minimally invasive surgeries. For example, while a large incident surface area is necessary to meet fluence requirements, a 6-mm diameter fused fiber bundle is too bulky to be attached to surgical tools, and in most cases, it would be larger than the surgical tool itself. Thus, a method to deliver light to the surgical site to simultaneously visualize vessels and the tool tip remains as a significant challenge despite the previously described advances. To address this particular challenge, this article explores the use of multiple fibers surrounding the tool tip to achieve the energy and fluence requirements for safe visualization of real blood. Our primary objectives are to determine how many fibers are necessary and to investigate their optimal spacing and placement relative to a real surgical drill. To the authors’ knowledge, no existing interventional photoacoustic applications address these important challenges of designing and optimizing a light delivery system to surround surgical tools.

## 2 Theory

**Fig. 1:**
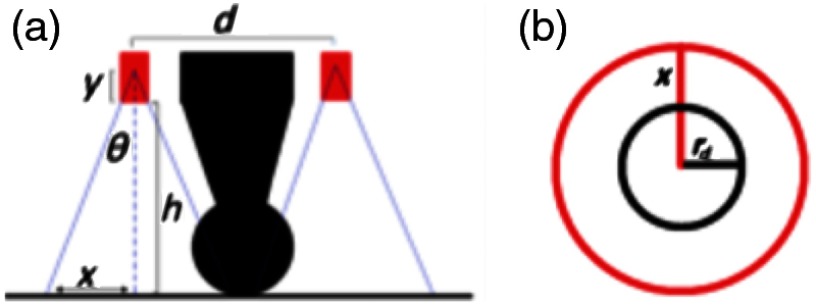
Geometry used to derive conical approximation of spot size showing views from (a) the side profile of the drill tip and tool shaft and (b) the detector surface touching the drill tip.

The laser spot size expected when multiple fibers surround a surgical drill with a spherical drill tip and generate a uniform light profile on the tissue surface may be calculated with geometrical optics, which assumes that a conical light profile is emitted from each optical fiber. This approximation predicts the total beam size. In the far-field approximation, it is assumed that the propagated beam has a constant intensity, and every ray hits the detector surface. The numerical aperture (NA) of the optical fiber is represented by NA. The fiber’s core diameter determines the distance, y, between the apex of the conical light profile and the fiber tip, whereas the distance between the fiber tip and the detector surface is h, as shown in Fig. 1. The distance between the center of opposing fibers is d, whereas x represents the radius of the conical profile on the detector surface, as shown in Fig. 1. The variables y and x can be determined from geometrical optics, where $y=\frac{\text{fiber core radius}}{\tan\;\theta }$, x=(h+y)tan θ, and $\theta =n\;\sin^{-1}\;\mathrm{NA}$, where n is the index of refraction (which is equal to 1, assuming that the light is propagating in air prior to hitting the tissue surface). This derivation resulted in the following equation for the maximum area in the far-field region:

$$A_{\text{far}}=\pi {\left(\frac{d+2x}{2}\right)}^{2}.$$ (1)

We used this far-field approximation to determine the maximum possible spot size and to compare this approximation to an actual photographed spot size.

A near-field approximation was calculated to predict the size of the torus formed when light is blocked by the drill, which is relevant when the drill is touching the tissue surface. In this approximation, $r_{d}$ represents the drill tip radius, and x is the same distance determined through the far-field calculation (i.e., the radius of the conical profile on the detector surface), resulting in the following equation for the near-field region:

$$A_{\text{near}}=\pi (4x^{2}-r_{d}^{2}).$$ (2)

**Fig. 2:**
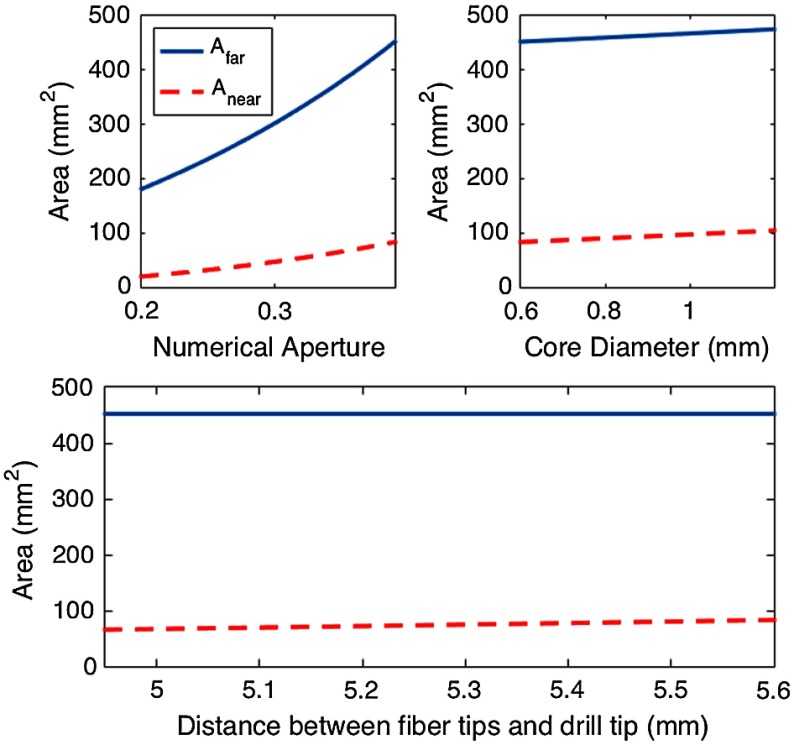
The theoretical near-field and far-field surface area approximations as functions of NA, fiber core diameter, and the distance that the fibers are set back from the drill tip. These plots are based on the actual drill geometry shown in Fig. 3 with a constant distance of h=20.1  mm from the fiber tips to the detector surface when measuring $A_{\text{far}}$, whereas $A_{\text{near}}$ represents measurements calculated with the drill tip touching the detector surface as shown in Fig. 1(a). Unless otherwise noted, the NA is 0.39, the fiber core diameter is 0.6 mm, and the fibers are set back a distance of 5.6 mm from the drill tip.

**Fig. 4:**
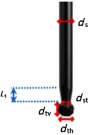
Actual drill geometry: drill shaft diameter $(d_{s})=2.37\;\mathrm{mm}$, drill shaft diameter after tapering $(d_{\mathrm{st}})=1.88\;\mathrm{mm}$, drill tip vertical diameter $(d_{\mathrm{tv}})=2.40\;\mathrm{mm}$, drill tip horizontal diameter $(d_{\mathrm{th}})=2.89\;\mathrm{mm}$, and length of taper $(L_{t})=3\;\mathrm{mm}$.

In addition to predicting spot sizes, Eqs. (1) and (2) may be used to calculate how fixed parameters (such as the NA, the fiber core diameter, and the distance that the fiber tip is set back from the drill tip) affect the overall spot size, as shown in Fig. 2. These plots are based on the actual drill geometry shown in Fig. 4 with a constant distance of h=20.1  mm from the fiber tips to the detector surface when measuring $A_{\text{far}}$, whereas $A_{\text{near}}$ represents measurements calculated with the drill tip touching the detector surface as shown in Fig. 1(a). These theoretical surface area approximations increase monotonically with both NA and fiber core diameter, while the distance that the fiber is set back from the drill causes up to $17\;{\mathrm{mm}}^{2}$ variation in the near-field approximations over the 4.95- to 5.60-mm range shown in Fig. 2. The theoretical far-field area is not affected by the distance the fiber is set back from the drill tip because the parameter h is held constant, and it represents the distance of the fiber from the detector surface. Thus, when the fiber is set back farther, the detector surface moves closer with this constraint, and the overall beam size on the detector surface does not change.

## 3 Methods

### 3.1 Monte Carlo Light Propagation Simulations

**Fig. 3:**
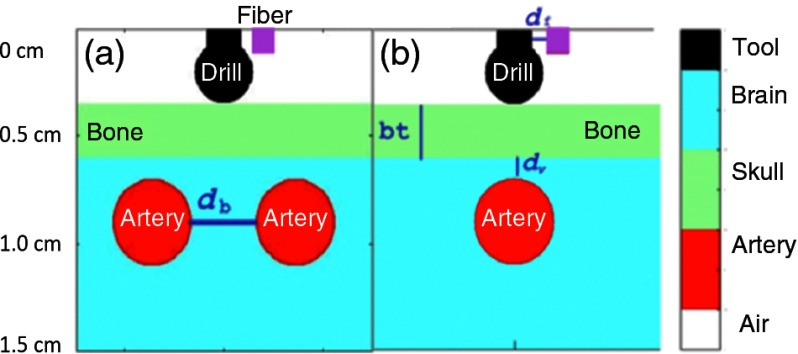
Monte Carlo Simulation diagram. (a) Two vessel simulation. The variable in this simulation was the distance between two arteries ($d_{b}$). (b) Single vessel simulation. The variables in this simulation were bone thickness (bt), distance between artery and bone ($d_{v}$), and distance between the fiber and the drill shaft ($d_{f}$).

Monte Carlo simulations^12^ were implemented to understand how the fluence seen by the arteries changes with respect to: (1) bone thickness, (2) distance between the artery and the bone, (3) distance between the light source and the drill shaft, and (4) distance between two arteries, i.e., the variables bt, $d_{v}$, $d_{f}$, and $d_{b}$, respectively, in Fig. 3. This information provides insight into potential artery visibility in a photoacoustic image. The Monte Carlo simulation traces the optical path from the light source in three-dimensional (3-D) space, voxel by voxel, also taking the optical properties for blood, bone, and brain matter into account, as well as those of the tool. The corresponding tissue and tool properties that we used in our simulation are summarized in Table 1.

**Table 1:** Optical properties of the tissue used in the Monte Carlo light propagation simulations.

Tissue	$\mu_{a}$ (${\mathrm{cm}}^{-1}$)	$\mu_{s}$ (${\mathrm{cm}}^{-1}$)	g (${\mathrm{cm}}^{-1}$)
Tool	2000	1000	0.9
Brain	2.3057	181.5859	0.9
Skull	0.1154	281.9549	0.9
Artery	230.5427	93.9850	0.9
Air	0.001	10.0	1.0

The simulations were split into two scenarios: single vessel and two vessels, as seen in Fig. 3. In both simulations, the drill was modeled with a spherical drill tip of diameter 2.9 mm connected to a cylindrical drill shaft of diameter 2.4 mm. This is an approximation of the actual drill tip geometry shown in Fig. 4. The metallic drill contacted the bone surface, and a single light source was placed next to the drill shaft, set 4.95-mm back from the drill tip. Note that this differs from the actual distance that the fiber was set back because the drill geometry was simplified for this study. The minimal effect of the fiber set back distance on the incident surface area is shown in Fig. 2 (bottom) for multiple optical fibers that surround the surgical drill. However in this study, only one fiber is attached to the surgical drill, thus the difference in fiber set back distances is expected to be negligible with respect to the final design.

For the single vessel simulations, the artery was simulated with a diameter of 4 mm and a length of 9 mm, and it was positioned directly below the drill. The expected diameter of an internal carotid artery is 3.7 to 8 mm,^13^^,^^14^ and the simulated artery is within this range. Though the drill placement directly above the artery may seem counterintuitive, it demonstrates that the system will work in the worst-case scenario, if the surgeon is in danger of damaging the internal carotid artery. The bone thickness was varied from 0 to 8 mm, the distance between vessel and bone was varied from 0 to 5 mm, and the distance between the source fiber and the drill shaft was varied from 0 to 5 mm. Only one parameter was varied at a time, otherwise, the bone thickness, fiber distance, and vessel distance (bt, $d_{f}$, and $d_{v}$, respectively, in Fig. 3) were held constant at 2.5, 1.25, and 1 mm, respectively.

For the two vessel simulations, the arteries had the same dimensions as the single vessel simulation, and they were positioned parallel to each other and equidistant from the drill. For these simulations, the distance between two internal carotid arteries [$d_{b}$ in Fig. 3(a)] was varied from 0 to 8 mm. The bone thickness, fiber distance, and vessel distance (bt, $d_{f}$, and $d_{v}$, respectively, in Fig. 3) were held constant at 2.5, 1.25, and 1 mm, respectively.

The output of these simulations was an image that displayed the normalized fluence in units of $\log_{10}({\mathrm{cm}}^{-2})$. The average normalized fluence was found by taking the sum along the artery surface closest to the bone then dividing by the artery’s length.

### 3.2 Zemax Ray-Tracing Simulations

Zemax simulations (Zemax LLC., Kirkland, Washington) were employed to model a metal drill acting as an absorber that blocked light from reaching the bone surface. The drill had a spherical drill tip of diameter 2.9 mm, and a drill shaft diameter of 2.37 mm. The tapering from 2.37 to 1.88 mm was taken into account in this simulation. The fibers were modeled as glass core and cladding, and they were set back at a distance of 5.6 mm from the drill tip. The core and cladding had the same index of refraction as the commercially available fibers we used for the prototype described in Sec. 3.3.

The goal of these simulations was to determine the number of fibers required for our light delivery system. Thus, the number of fibers was varied from 1 to 10, and we identified the threshold where the multiple beams incident upon the bone overlapped enough to make one individual beam rather than form multiple hot spots. Smoothing was applied to the beam profile. The output was taken in position space, so that spot size could be measured with a pixel-to-millimeter conversion factor. To qualitatively determine whether or not a spot was uniform, the images were exported to MATLAB^®^ (MathWorks, Natick, Massachusetts) and thresholded. The threshold was set at 1/e times the peak intensity. If the pixels that are within 1/e of the peak intensity of the image form a complete torus, then we considered this to indicate uniformity at the detector surface. The 1/e beam profile was used for thresholding because the American National Standards Institute (ANSI) layer safety limits are based on this measurement.^11^

### 3.3 Light Delivery System Design Requirements

We built a light delivery prototype based on design requirements that were determined from the simulation results. The first design requirement is that seven or more fibers are necessary to achieve the desired beam profile as demonstrated in more detail in Sec. 4.2. Second, the fibers should be equally spaced and held 2 mm away from the drill shaft, as determined in Sec. 4.1. A commercially available 1-to-7 splitter was utilized to meet these requirements. The fiber was modified by cleaving the SMA connectors from the seven-fiber fan-out end and exposing 2 cm of the fiber jacket and 1 cm of the fiber cladding. The fibers were then polished for a flat cleaved finish. The fibers were held 2 mm away from the drill and equally spaced using a custom 3-D printed part.

Although we decided to constrain our design to a commercially available fiber with an NA of 0.39 and a fixed fiber core diameter of 600  μm, we note that changing the NA and core diameter would likely alter the optimal results that guided our design requirements as demonstrated in Fig. 2 (e.g., the optimal number of fibers is indirectly related to the incident surface area that monotonically increases with an increase in NA and core diameter). However, any changes to these constraints can be explored with the same methods reported in Sec. 4 to achieve new design requirements. We also assume that the fiber axes and drill axis would be parallel to each other and that the relationship between laser light and drill tip during the drilling process would have negligible effects on the results that we obtained.

### 3.4 Beam Profiler

An Edmund Optics (Barrington, New Jersey) USB 3.0 beam profiler was used to measure the beam profile output from our design. The fiber was coupled to a Quantum Ultra 1064 nm Nd:YAG pulsed laser (Quantel Bozeman, Montana). The beam profiler has a built-in distance of 20.1 mm between the sensor and the outer face of the neutral density filter. This limits the sensor to capturing the far field beam profile and excludes our ability to measure the near-field profile with this device. The white light flashlamp output (which was coincident with the laser output) was used to determine the beam profile to avoid damaging the sensor with the high power output from the Nd:YAG laser. The primary purpose of these experimental beam profile measurements was to compare them to simulation results for assessment of ANSI laser safety requirements.

### 3.5 Photoacoustic Imaging Experiment

Our photoacoustic imaging system consisted of an Alpinion ECUBE12R ultrasound system, Alpinion L3-8 linear transducer (3 to 8 MHz bandwidth), and the light delivery system described in Sec. 3.3, coupled with either a Quantum Ultra 1064 nm Nd:YAG pulsed laser or a Phocus Mobile Laser (Opotek, Carlsbad, California). The Quantum Ultra laser was pulsed at a rate of 20 Hz with a pulse length of 7 ns and a pulse energy of 0.75 mJ. The Phocus Mobile laser was programmed to emit 790-nm light, which was pulsed at a rate of 10 Hz with a pulse length of 5 ns and a pulse energy of ∼15  mJ. It was helpful to use two different laser setups to evaluate the ability of our light delivery prototype to work under different laser conditions.

**Fig. 5:**
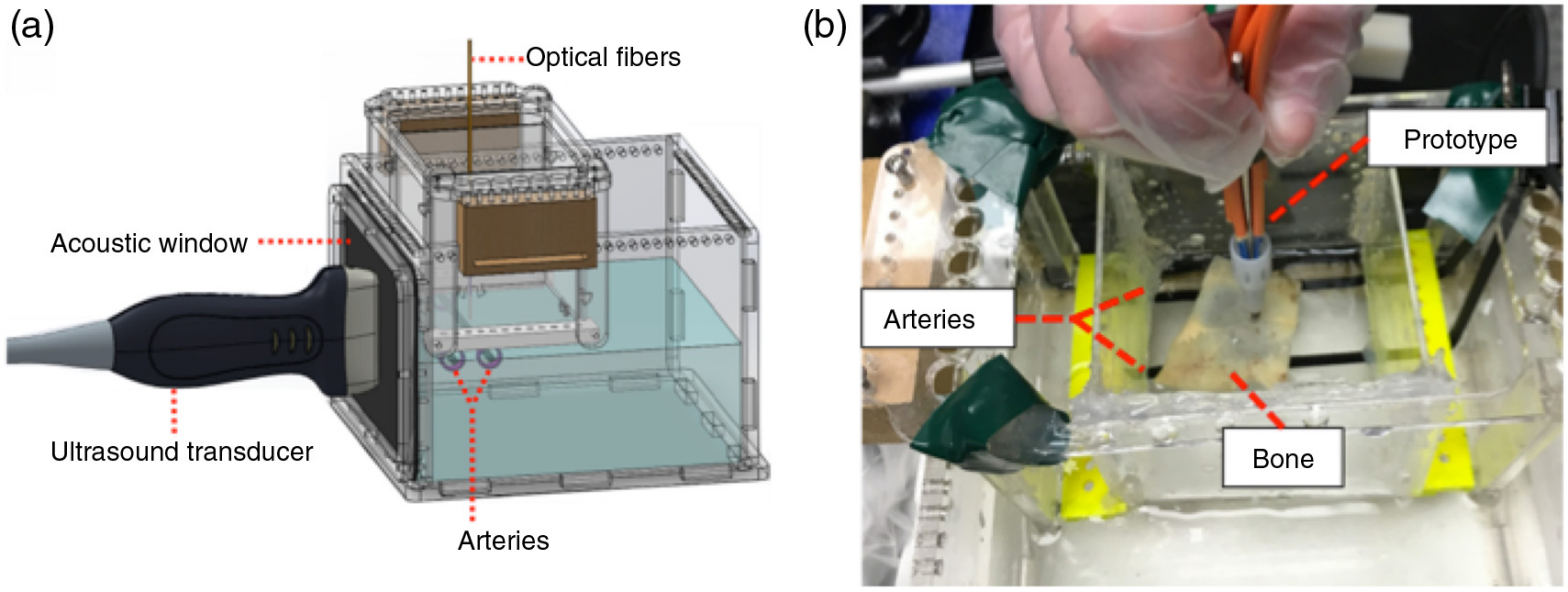
(a) Solid model of phantom and (b) experimental setup with light delivery prototype used to image through a cadaveric bone specimen.

Our photoacoustic system was used to image a phantom containing two rubber rods that mimicked blood vessels. Our experimental setup is shown in Fig. 5. The phantom consisted of an acrylic container with an open bottom nested inside a larger acrylic container containing an acoustic window. This larger container was filled with water. Holes along the sides of the smaller container allowed for adjustment of the rubber rod placement.^15^

These blood vessel-like targets were imaged with and without human cadaveric bone specimens^16^ placed between the drill tip and the vessels, as shown in Fig. 5. The Quantum Ultra laser was used for imaging when bone was absent, whereas the Phocus Mobile laser was used for imaging when bone was present. Photoacoustic images were acquired with the tool tip located between the two vessels. A synchronized video showing the fiber motion relative to the resulting real-time photoacoustic images was also created (Video 1). A conventional delay-and-sum beamformer was used to display all photoacoustic images.

## 4 Results

### 4.1 Monte Carlo Simulation Results

**Fig. 6:**
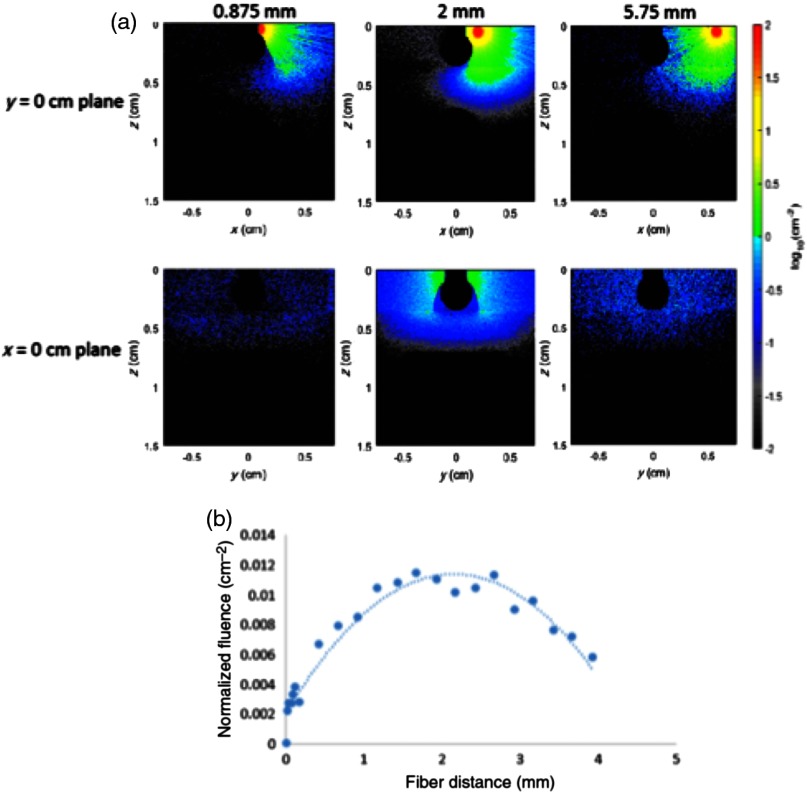
The distance of the fiber from the drill shaft alters the normalized fluence distribution. The images (a) display the normalized fluence when the fiber is located at distances of 0.875, 2, and 5.75 mm from the drill shaft (as indicated above each image), while the plot (b) shows measured data points along the artery surface as a function of multiple fiber distances. The quadratic curve $F_{N}=-0.002d_{f}^{2}+0.0086d_{f}+0.0021$ was fit to the data points.

When the distance between the source and the drill shaft ($d_{f}$) was varied, the resulting normalized fluence, $F_{N}$, can be represented by a quadratic function: $F_{N}=-0.002d_{f}^{2}+0.0086d_{f}+0.0021$, as shown in Fig. 6. This plot and the corresponding example images indicate that much of the light is blocked by the drill when the fiber is too close to the drill shaft, but when the fiber is too far, the light does not adequately illuminate the underlying vessel. The optimal distance was found to be 2 mm. This result was incorporated into the Zemax physical optics propagation simulations.

**Fig. 7:**
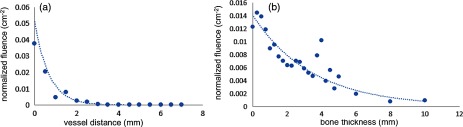
Normalized fluence as a function of (a) vessel distance and (b) bone thickness.

As expected, fluence decreases as bone thickness and vessel distance increase, as seen in Fig. 7. When the artery is ∼3  mm away from the bone, fluence is approximately zero, and when the bone thickness is 5 mm or greater, the normalized fluence seen by the artery is minimal ($F_{N}<0.006\;{\mathrm{cm}}^{-2}$), indicating that the vessel is unlikely to be visible in a photoacoustic image.

**Fig. 8:**
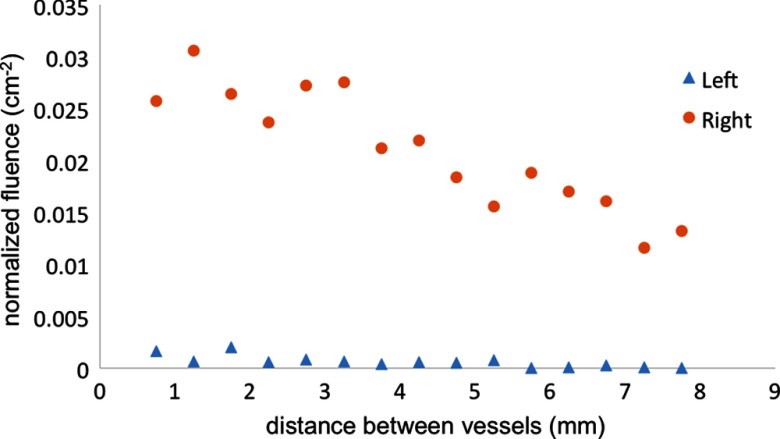
Normalized fluence as a function of the distance between two arteries.

The two-vessel simulation showed that there is a significant difference in fluence between two vessels if only one source fiber is used as shown in Fig. 8. The fluence seen by the vessel farthest from the fiber is approximately zero. This result shows that it is unreasonable to use one fiber in our design because it would be difficult to visualize two arteries simultaneously and because the asymmetry would not provide accurate information about vessel proximity if approaching an artery from the fiberless side of the tool.

To interpret these results in terms of fluence rather than normalized fluence, an input energy of 25 mJ was arbitrarily chosen. Based on a bone thickness of 2.5 mm, vessel distance of 1 mm, and optimal fiber distance of 2 mm, the normalized fluence seen by the bone surface was measured by averaging the normalized fluence values found along the bone center line located directly above the artery. The corresponding measurement was repeated for the artery surface. We used the following expression for fluence:

$$\text{Fluence}=E\times F_{N},$$ (3)

where E is the laser output energy. The fluence at the bone surface was $9.7\;\mathrm{mJ}/{\mathrm{cm}}^{2}$, whereas the fluence at the artery surface was $0.3\;\mathrm{mJ}/{\mathrm{cm}}^{2}$. These results indicate that the exposed bone surface experiences 32 times more fluence than the underlying artery.

### 4.2 Zemax Results

**Fig. 9:**
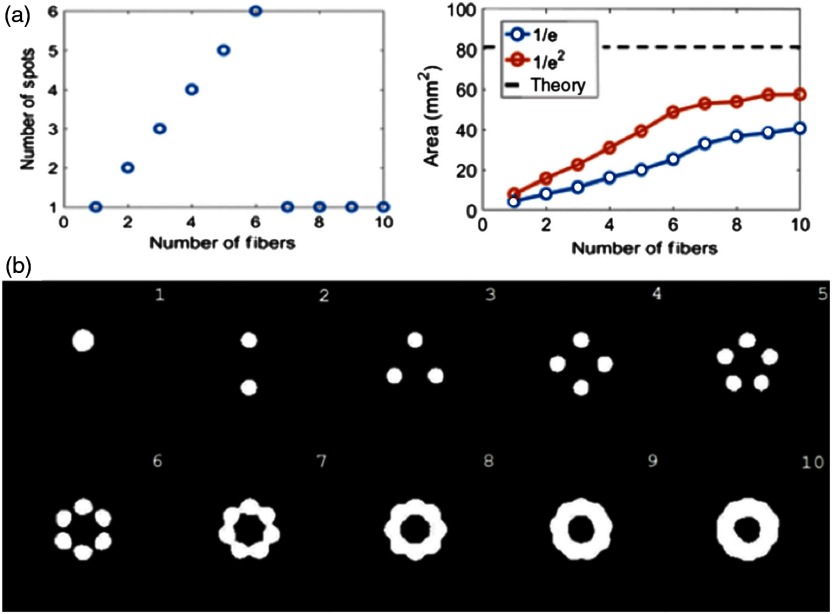
(a) Number of spot sizes observed and 1/e area of the spot sizes as a function of the number of fibers surrounding the drill. (b) Images showing the 1/e thresholding used to calculate area as the number of fibers increased. The beam profiles converge with seven or more fibers.

Zemax simulations were implemented to investigate the minimum number of fibers required to surround the surgical drill, which is tied to the laser spot size obtained with more than one source fiber. The incident laser spot size increased as the number of fibers increased, and the number of spots eventually transformed from creating multiple hot spots to creating a single beam, as shown in Fig. 9. A single uniform beam was formed with seven or more fibers for a NA of 0.39 and a core diameter of 600  μm. The measured area results were compared with the near-field area approximation of the total beam area, as described by Eq. (2) (i.e., $83.2\;{\mathrm{mm}}^{2}$ at the bone surface in contact with the drill tip).

**Fig. 10:**
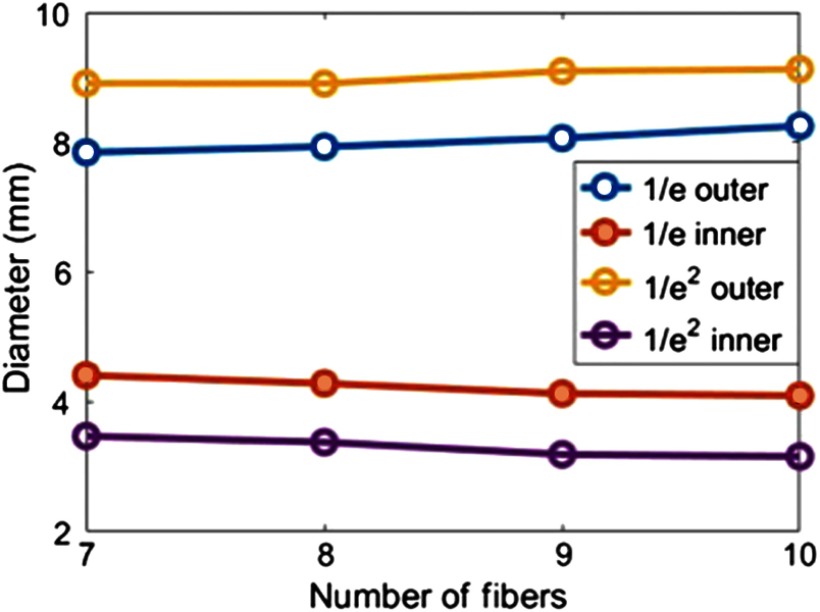
Inner and outer diameters of 1/e and $1/e^{2}$ beam profiles detected on a planar surface that is coincident with the drill tip and orthogonal to the drill axis.

A related measurement for the increase in spot size is beam diameter rather than surface area. Note that as the number of fibers increases, the beam’s outer diameter increases, whereas the inner diameter decreases. This can be visualized qualitatively from the thresholded images in Fig. 9, and it can be quantified based on the 1/e and ${1/e}^{2}$ beam diameters, which were measured for 7 to 10 fibers in Fig. 10. This measurement was implemented by determining the threshold boundaries along the beam’s center line and then calculating the corresponding beam diameters.

The near-field Zemax beam profile results obtained when the drill tip is touching the bone surface were compared with the near-field theoretical approximation derived in Sec. 2. The near-field approximation estimates a fixed inner diameter of 2.9 mm for the total beam size, based on the diameter of the spherical drill tip (i.e., $r_{d}$). The Zemax simulation results show that the inner diameter can be larger than 2.9 mm for less than 10 fibers, whereas the total beam inner diameter approaches that of the $1/e^{2}$ inner beam diameter with 10 fibers surrounding the drill, as evident in Fig. 10. When comparing these results with the Monte Carlo simulation results [i.e., Fig. 6 (top)], we note that the near-field inner diameter also depends on the distance between the light source and the drill shaft.

**Fig. 11:**
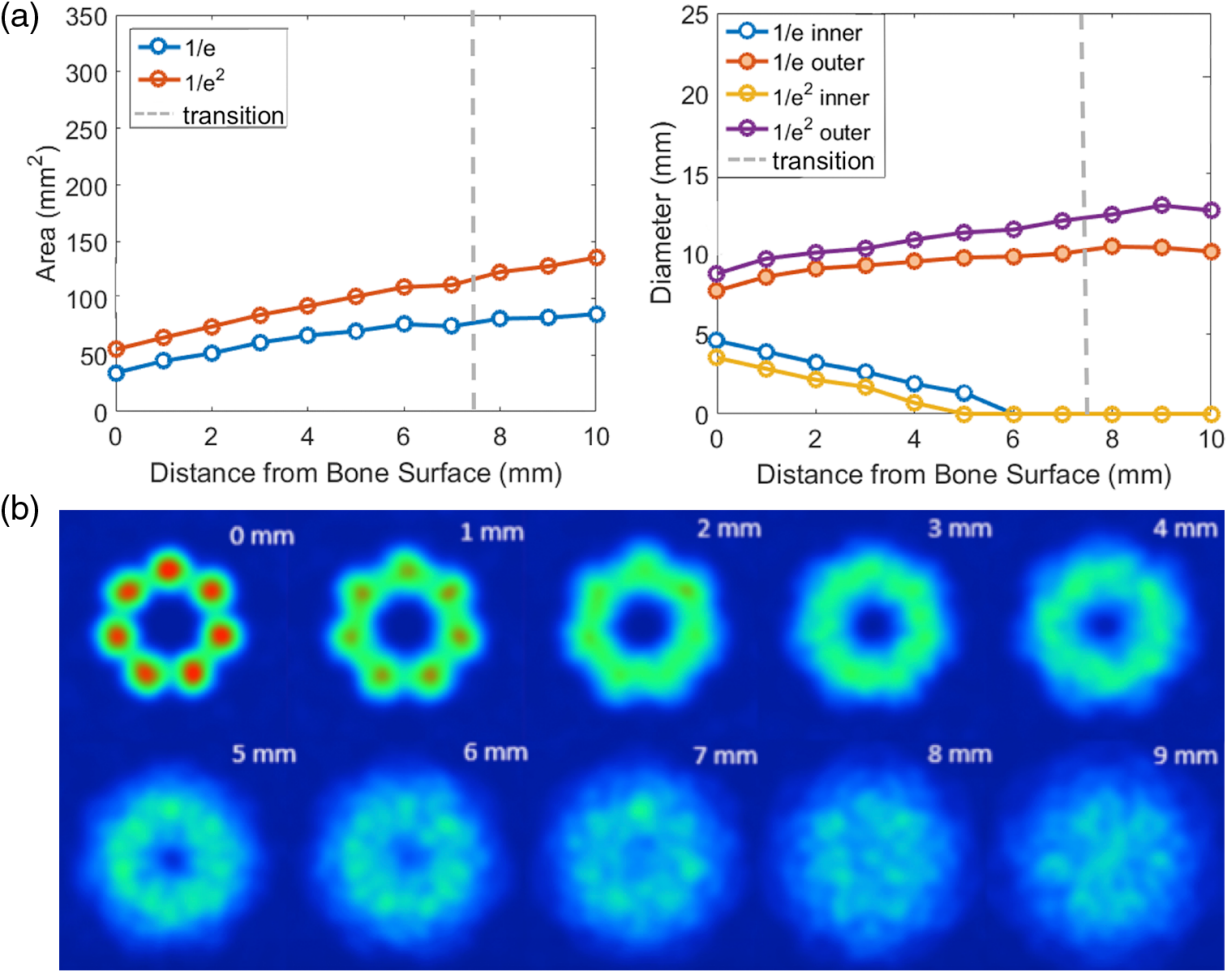
Near- and far-field beam profile results. (a) The plots show 1/e and $1/e^{2}$ beam profile areas and diameters as a function of the tool tip distance from the detector surface, which represents the bone that will be drilled. The dashed vertical line indicates the transition from near-field to far-field beam profiles (determined when the $1/e^{2}$ beam profile decreases to zero). (b) The pictures demonstrate this transition of the beam profile from a torus to a Gaussian as the distance between the drill tip and the surface increases from 0 mm to 9 mm.

Figure 11 shows that as the drill is moved away from the detector surface (which could represent the bone or tissue surface that blocks an underlying structure of interest), two important things happen. First, the spot size increases. This is expected based on basic trigonometry, but it is important for this design because it means that the field of view widens, and the fluence decreases. Second, the beam profile changes from a torus to a Gaussian beam, where it is most intense at the center, as seen in Fig. 11. This transition occurs at a distance of ∼12 to 13 mm from the fiber tips, which corresponds to ∼6 to 7 mm from the drill tip as shown in Fig. 11 (because the fibers are set back 5.6 mm from the drill tip).

### 4.3 Light Delivery System Prototype

**Fig. 12:**
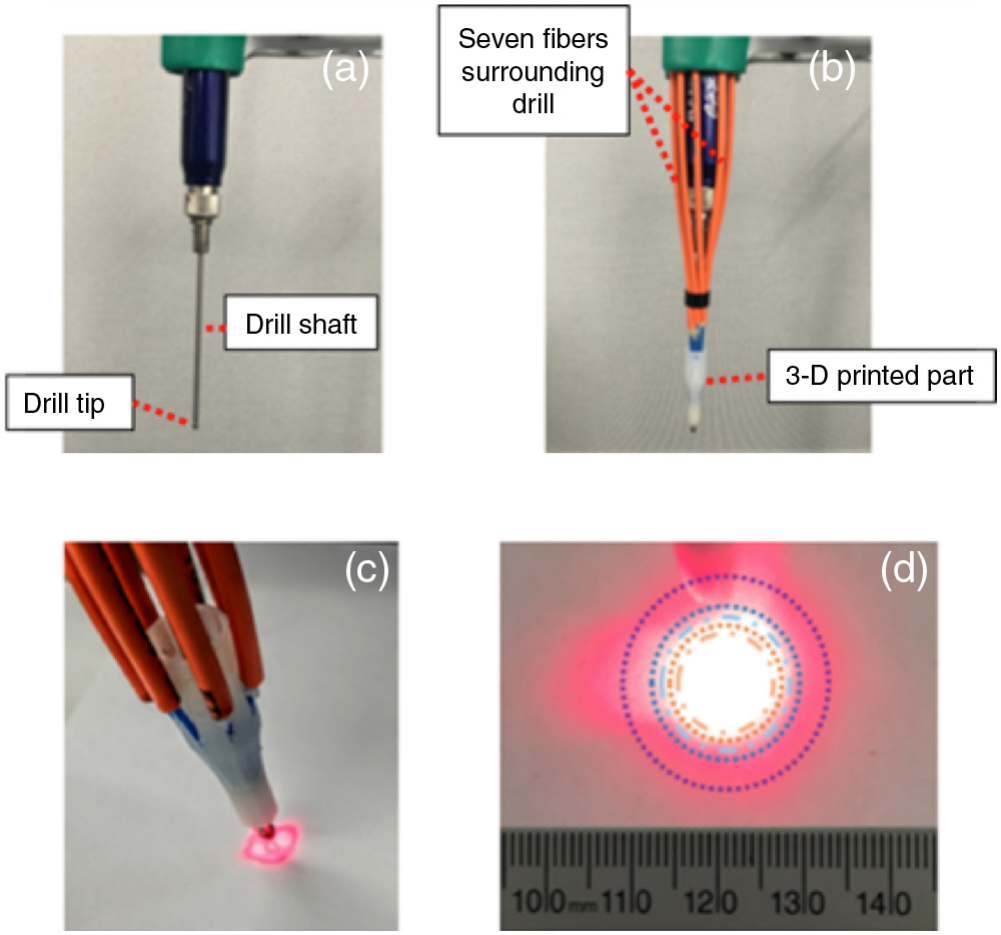
(a) Surgical drill without attachments, (b) light delivery prototype with optical fibers surrounding the drill and secured into the 3-D printed part, (c) a 635-nm laser light coupled with this light delivery system shows the near-field spot size, (d) the resulting far-field laser spot size at a distance of ∼20  mm from the fiber tips, showing comparisons to theoretical, simulation, and experimental results (i.e., the rings from largest to smallest represent beam diameters measured based on the far-field theory, $1/e^{2}$ beam profiler and Zemax results, and 1/e beam profiler and Zemax results).

The simulation results provided design requirements for our light delivery system prototype, which are summarized in Sec. 3.3. The prototype consists of seven fibers that surround the drill and are held in place by a custom 3-D-printed part, as shown in Figs. 12(a) and 12(b). The near-field and far-field light profiles are displayed in Figs. 12(c) and 12(d), respectively, when 635-nm light is propagated through our prototype.

### 4.4 Comparing Spot Size

**Fig. 13:**
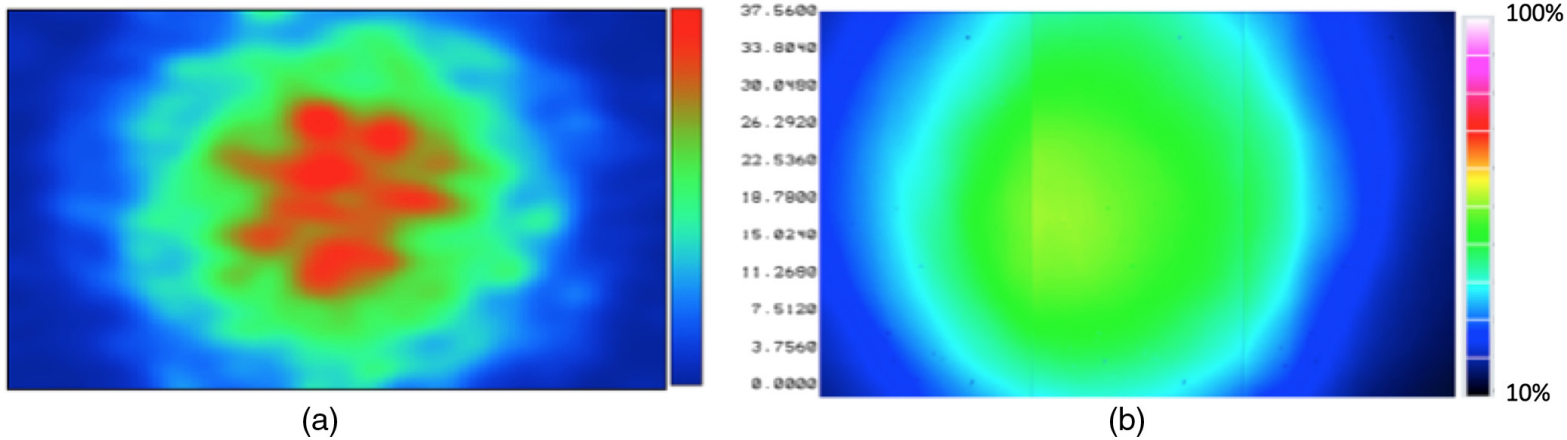
Beam profile 20.1 mm away from the fiber surface measured with (a) Zemax and (b) the beam profiler. The peak intensity is lower than 100% with the beam profiler result because data are not normalized. The dimensions of these images are 11.3  mm×18  mm.

**Fig. 14:**
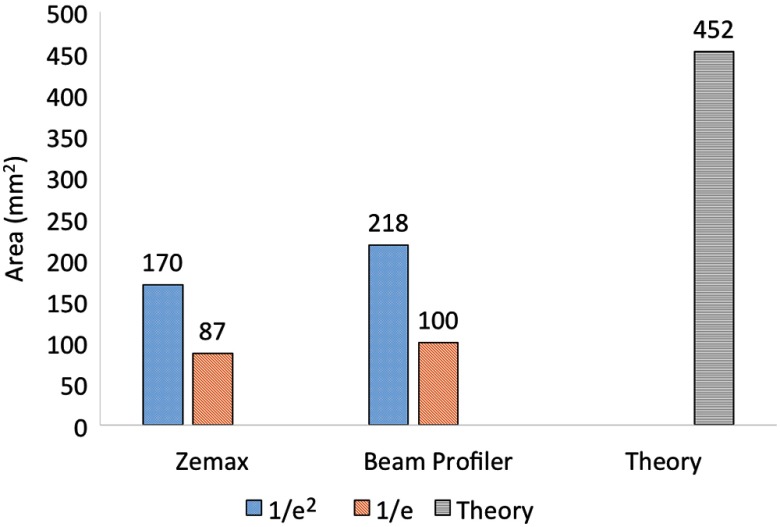
Measured 1/e and $1/e^{2}$ spot sizes at a distance of 20.1 mm away from the fiber surface. The theoretical approximation for the total beam size is shown for reference.

The spot size obtained with our prototype was approximated through Zemax ray-tracing simulations and experimentally measured with a beam profiler at a distance of 20.1 mm from the detector surface, as shown in Fig. 13. The 1/e and $1/e^{2}$ spot sizes were 87 and $170\;{\mathrm{mm}}^{2}$, respectively, for the Zemax simulations and 100 and $218\;{\mathrm{mm}}^{2}$, respectively, for the experimental results, as shown in Fig. 14. The corresponding far-field approximation was $452\;{\mathrm{mm}}^{2}$ at the same distance from the detector surface.

These quantitative results were qualitatively compared with the photograph of the beam profile shown in Fig. 12(d), by assuming a circular profile and converting the area measurements to their corresponding diameters. Each ring shows the diameter for one of the five quantitative results reported in Fig. 14. From outer to inner ring, we see the spot sizes obtained with: (1) the far-field theoretical approximation for the total beam diameter, (2) the beam profiler at 1/e threshold, (3) the Zemax simulations at 1/e threshold, (4) the beam profiler at $1/e^{2}$ threshold, and (5) the Zemax simulations at $1/e^{2}$ threshold. Qualitatively, it appears that the $1/e^{2}$ and 1/e spot sizes obtained from the experimental and simulation results tend to approximate the 635-nm light in the photograph of Fig. 12(d) with reasonable accuracy, whereas the theoretical approximation for the total beam diameter generally seems to trace the outermost edges of the beam profile.

### 4.5 Photoacoustic Imaging with the Prototype Light Delivery System

**Fig. 15:**
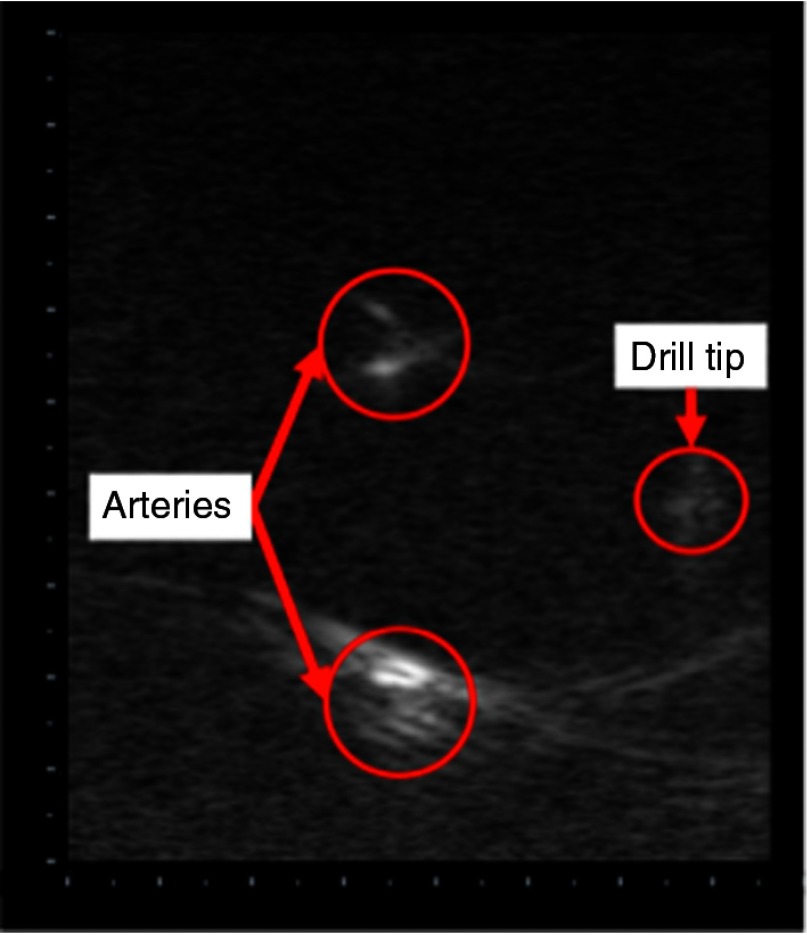
Photoacoustic image obtained with our multifiber light delivery system design. The total vertical depth is 4.5 cm, and each mark depicts a spacing of 0.25 cm. A video (Video 1) showing synchronized fiber motion and real-time photoacoustic images are included as a multimedia file. The video starts with the prototype outside of the water. Photoacoustic signals appear on the left as the tool is inserted in the water and navigated around the two vessels (Video 1, MPEG 4.2 MB [URL: http://dx.doi.org/10.1117/1.JBO.22.4.041011.1]).

A photoacoustic image was taken with our light delivery prototype using the setup shown in Fig. 5. The resulting image obtained without bone appears in Fig. 15. The image is oriented such that the ultrasound probe is located at the top of the image. Note that both the vessel boundaries and the drill tip are visible in a single image. A synchronized video showing the fiber motion, and resulting real-time photoacoustic images is included as a multimedia file (Video 1). The photoacoustic signals from the drill tip are clearest when the tip is located within the image plane.

**Fig. 16:**
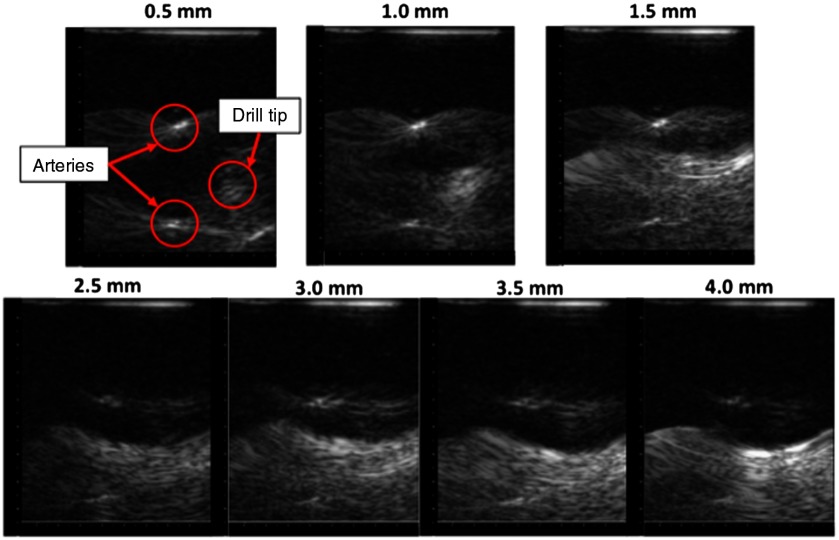
Photoacoustic images obtained when bone is placed between the drill tip and vessels, as shown in Fig. 5. The drill tip is consistently located between the two vessels and becomes increasingly difficult to visualize as bone thickness increases, particularly when the drill tip is not perfectly aligned with the image plane. It also appears that the thicker bone samples (e.g., 4 mm) are visible in the photoacoustic image. The total vertical depth of each image is 4.5 cm, each mark depicts a spacing of 0.25 cm, and all still images are shown with 60-dB dynamic range. A video (Video 2) showing real-time photoacoustic images obtained in the presence of 1.5-mm-thick bone is included as a multimedia file; images in the video are displayed with 30-dB dynamic range (Video 2, MPEG 347 kB [URL: http://dx.doi.org/10.1117/1.JBO.22.4.041011.2]).

Cadaveric bone specimens ranging in thickness from 0.5 to 4.0 mm were individually added to this experimental setup by placing the bone on top of the vessels and pressing down on the bone with the drill tip. The resulting images are shown in Fig. 16 with the bone thickness indicated at the top of each image.

As the bone thickness increased, we observed three important changes. First, the acoustic signals from the drill tip became more scattered, making the drill tip less distinguishable in static images, as shown in Fig. 16 (although the drill location relative to the vessels is evident in the real-time images, particularly when the drill tip is aligned with the image plane, as demonstrated in Video 2). The dynamic range of the photoacoustic images in Video 2 was reduced to 30 dB to enhance the visibility of the photoacoustic signals of interest. In general, image settings such as dynamic range may be optimized to enhance drill tip visualization, which would be necessary to maintain optimized amplitude-based images (e.g., delay-and-sum beamformed images) based on our second observation that the vessel contrast decreases as bone thickness increases. This second observation is evident given the fixed dynamic range of the images shown in Fig. 16, and it is consistent with previous results that quantify the relationships among bone thickness, light transmission, and target contrast.^16^ Third, it appears that the thicker bone samples (e.g., 4 mm) are visible in the photoacoustic image, which is also consistent with previous findings.^2^ This bone visibility could potentially compensate for the poor visibility of the drill tip at the higher bone thicknesses, as only the portion of the bone illuminated by the light delivery system is visible in the photoacoustic image.

## 5 Discussion

We successfully designed and built a light delivery system prototype based on the integration of Monte Carlo simulations, Zemax simulations, beam profiler results, and theoretical calculations. This is the first multifiber design for an interventional photoacoustic system to visualize a surgical tool tip simultaneously with targets (e.g., blood) for guiding surgeries. With this design, we achieved photoacoustic images that simultaneously visualize the blood vessel boundaries, the drill tip, and in some cases, bone in a single frame, as shown in Figs. 15 and 16 and in Videos 1 and 2. The varying laser conditions that we tested demonstrate that our light delivery prototype is operable under multiple conditions.

Because the use of multiple fibers surrounding the tool tip increases the maximum achievable spot size compared with the fused fiber bundle approach,^10^ we can now use a higher energy input to make photoacoustic images (assuming that the average energy of the merged Gaussian beams from each individual fiber will not exceed ANSI laser safety limits). Based on a conservative 1/e estimation of spot size, the merged beam profile area ranges from 42 to $76\;{\mathrm{mm}}^{2}$, depending on distance from the detector surface, as seen in Fig. 11. This result can be interpreted in terms of an allowable output energy range for comparison with the previous fiber bundle approach.^10^ For example, when visualizing blood at a fluence limit of $25\;\mathrm{mJ}/{\mathrm{cm}}^{2}$ (which is less than the ANSI limits of 30 to $100\;\mathrm{mJ}/{\mathrm{cm}}^{2}$ for skin for the 790 and 1064 nm wavelengths used in our photoacoustic experiments), the $\;42\;to\;76\;{mm}^{2}$ range of spot sizes corresponds to an input energy range of 10 to 19 mJ.

Considering that at least 1.2 to 6 mJ is required to visualize blood through bone thicknesses ranging from 0 to 2 mm,^10^ the results in this article indicate that we can potentially use higher energies without increasing patient risk, particularly when the bone is thicker than 2 mm. According to Monte Carlo simulation results (Fig. 7), bone thicknesses up to 4 or 5 mm would require higher energies to increase the fluence to the blood vessel. Although our experimental results demonstrated that the scattering that occurs as bone thickness increases causes the boundaries of the tool tip to become less distinguishable in an otherwise aqueous environment, a tool tip located at the center of the two vessels is still discernable at the higher bone thicknesses. Alternatively, at these higher bone thicknesses, the bone sample becomes visible in the photoacoustic image and could potentially serve as a surrogate for the tool tip location (because only the portion of the bone illuminated by the light delivery system is visible in the photoacoustic image). In addition, the Monte Carlo simulation results demonstrate that the bone surface may experience up to 32 times higher fluence than the underlying vessel and surrounding tissue, which is potentially responsible for the bone visibility in the photoacoustic image and additionally advantageous for not damaging underlying tissue at these higher energies. If necessary, damage to the bone surface at these higher energies may be acceptable considering that the bone will be destroyed throughout the drilling process.

This paper explored three different approaches to determine the expected laser spot size that would be obtained with our prototype, as shown in Fig. 12. Although the three approaches provide different measurements, when approximated to the nearest 100th, both the Zemax and the beam profiler results provide 1/e and $1/e^{2}$ spot sizes of 200 and $100\;{\mathrm{mm}}^{2}$, respectively. Potential sources of error when comparing these measurements include the different wavelengths that were used for each measurement and subtle differences between the distances that the fiber’s distal end was set back from the drill tip. In addition, the far-field theoretical approximation ($452\text{-}{\mathrm{mm}}^{2}$ total area) accurately predicts that the entire beam is larger than these simulation and experimental results, and the photograph outlining the corresponding diameter shows that the theoretical result reasonably encompasses the total optical beam.

Although the theoretical results cannot be directly compared with the simulation and experimental results, because they are measuring different beam sizes (i.e., total diameter versus the diameter at 1/e or $1/e^{2}$ times the maximum beam amplitude), the theory can potentially be related to the 1/e and $1/e^{2}$ area measurements through factors of 4.3 to 4.9 and 1.9 to 2.5, respectively, for the specific cases explored in this article. Appropriate factors for other cases may be determined by relating theory to simulations for a new set of fixed design parameters.

We note that the custom 3-D printed plastic part used to hold the fibers in place could potentially act as a mechanical bushing that enables drill rotation and operation while the multifiber locations remain stationary. In the future, this 3-D printed part will be attached to the stationary handle of the surgical drill for testing while the drill is in motion. Future work will additionally include testing this design with real blood vessels and other targets of interest (e.g., nerves) for multiple photoacoustic-guided interventional applications.

Although translation of this technology into clinical practice for neurosurgical guidance requires some degree of initial testing on a whole skull model, there are other surgical applications that do not require this skull model and thus present additional benefits for the proposed multifiber light delivery system design. Hence, this article documents a significant step for the present stage of our technology. Improvements to the phantom model will be the focus of future work, but we do not expect that these improvements will affect our major conclusions regarding the design requirements for our new light delivery system and the use of simulation tools to assist with defining these requirements for a range of surgical instruments.

## 6 Conclusion

We have reported our success with designing, building, and preliminary testing of a multifiber light delivery system to surround surgical tool tips. In particular, the design reported in this paper is optimized for a neurosurgical drill. For a 2.9-mm spherical drill tip, the optimal fiber distance from the 2.4-mm drill shaft was identified as 2 mm. At this optimal distance, the optical profile merges with seven or more fibers. The increased spot size with a 1-to-7 fiber splitter decreases fluence and enables higher energies within safety limits. The methodology used to obtain these results may be applied to design and build custom multifiber light delivery systems for an entire suite of surgical tools.
